# Does selection in a challenging environment produce Nile tilapia genotypes that can thrive in a range of production systems?

**DOI:** 10.1038/srep21486

**Published:** 2016-02-19

**Authors:** Ngo Phu Thoa, Nguyen Huu Ninh, Wayne Knibb, Nguyen Hong Nguyen

**Affiliations:** 1 Research Institute for Aquaculture No.1, Dinh Bang, Tu Son, Bac Ninh, Vietnam; 2 Faculty of Science, Health, Education and Engineering, University of the Sunshine Coast, Maroochydore QLD 4558, Australia

## Abstract

This study assessed whether selection for high growth in a challenging environment of medium salinity produces tilapia genotypes that perform well across different production environments. We estimated the genetic correlations between trait expressions in saline and freshwater using a strain of Nile tilapia selected for fast growth under salinity water of 15–20 ppt. We also estimated the heritability and genetic correlations for new traits of commercial importance (sexual maturity, feed conversion ratio, deformity and gill condition) in a full pedigree comprising 36,145 fish. The genetic correlations for the novel characters between the two environments were 0.78–0.99, suggesting that the effect of genotype by environment interaction was not biologically important. Across the environments, the heritability for body weight was moderate to high (0.32–0.62), indicating that this population will continue responding to future selection. The estimates of heritability for sexual maturity and survival were low but significant. The additive genetic components also exist for FCR, gill condition and deformity. Genetic correlations of harvest body weight with sexual maturity were positive and those between harvest body weight with FCR were negative. Our results indicate that the genetic line selected under a moderate saline water environment can be cultured successfully in freshwater systems.

Understanding the interactions between genotype and environment (G × E) is of utmost importance in the design of selective breeding programs to develop improved strains that express optimal growth and adaptability in a wide range of environments. In a review of 24 studies in both farmed animals and model species, Falconer^1^ proposed that selection in a less favourable environment (i.e. antagonistic selection) may produce genotypes that perform well across a range of production systems. In contrast, selection under favourable environmental conditions (i.e. synergistic selection) may result in sensitive genotypes which do not thrive under stressed conditions. We have developed a genetic line of Nile tilapia under sub-optimal culture environment (i.e. moderately saline water of 10–20 ppt). Genetic evaluation of the line selected over five generations from 2008 to 2012 showed an increase in body weight in saline water of approximately 7% per generation (one generation was completed each year)^2^. However, it is not known if these increases in saline water are expressed in the freshwater production systems common in tilapia aquaculture. This information is necessary before the improved strain can be released into commercial production.

Several studies have examined the effect of G × E interaction by testing full- and half-sib families in varying conditions, such as between culture systems^3^,^4^,^5^, dietary protein levels^6^, animal *vs.* plant based protein diets^7^,^8^,^9^, feeding regimes and rearing schemes (high *vs.* low stocking density)and between diverse environmental parameters^10^. A synthesised results from the literature show that when the selection environments differ remarkedly from production systems, the G × E effect is statistically significant^5^. On the other hand, when the selected environment in the nucleus is close to commercial production, the interaction between genotype and environment may not be of biological importance^11^.

While many studies have investigated the traits of growth and survival, and their respective G × E interactions, as it relates to profit and income for aquatic species, less well understood are the traits of feed conversion ratio^12^, sexual maturity, fish morphology^13^,^14^ and their GxE effects. Further, morphological deformities generally have negative impacts on marketability and these characters (fitness related traits), but typically have not been included as part of the breeding objectives in most previous selection programmes. Abnormalities may be caused by inbreeding^15^, non-heritable factors such as diseases^16^, environmental disturbances^17^,^18^ as well as sub-optimal culture conditions^19^,^20^,^21^. Only a few studies have reported the existence of G × E effect for sexual maturity in aquatic animal species^22^,^23^ which is curious the important possibility that G × E interactions may cause a decline in reproduction. Similarly little is known about G × E interactions for fitness and resistance against diseases. Indeed, there is no information regarding quantitative genetic basis of gill diseases, morphological deformity and food conversion ratio (FCR) in tilapia. The question of whether selection in harsh environments permitted fast growth in benign conditions remains unanswered.

The principal objective of this study was to investigate whether selection for high growth rate in a challenging environment of medium salinity produces tilapia genotypes that can perform well in freshwater systems. In resolving this, we (i) examined the effect of genotype by environment interaction for new traits (sexual maturity, deformity, survival and FCR) in a Nile tilapia population undergoing five generations of selection for high growth in moderate saline environment (15–20 ppt); ii) estimated the genetic parameters for these traits together with gill related diseases and body weight; and (iii) deciphered phenotypic and genetic associations among traits recorded during the selection process (2007 to 2014).

## Materials and Methods

### Experimental animals

The fish used in this study originated from a genetic line which has undergone five generations of selection (2008–2012) for increased harvest body weight in moderately saline water (15–20 ppt) in Vietnam. During this period, a total number of 922 breeders (525 females and 397 males) were used to produce progeny with the average selected proportion of 4.43% in females and 3.48% in males each generation. Within- and between-family selection was practised based on estimated breeding values (EBVs) for body weight. The EBVs were estimated for all individuals in the pedigree using a statistical model that included the fixed effects of generation, sex and age and the random additive genetic effect of individual animal^2^. The number of selected individuals contributed by each family to subsequent generations was restricted and closely related matings among full- and half-sibs was avoided to control realized inbreeding. The accumulative level of inbreeding in the present population (0.51% per generation) was within the acceptable level (which is less than 1% per generation) in closed breeding nucleus^24^. The selection program resulted in a significant improvement in body weight withan average gain of approximately 7% per generation (one generation per year)^2^.

### Pond preparation and family production

Earthen ponds of various sizes (e. g. 50 × 40 × 1.2 m) were used for conditioning, breeding and nursing and were prepared following a standard protocol^25^. Briefly, ten days before the experiment, an inorganic fertilizer (triple super phosphate) was applied to each pond at a rate of 50 kg per hectare to stimulate the production of natural food sources (e.g. phytoplankton and zooplankton). Breeding to produce offspring for this study began in March 2014 and followed a period of conditioning of the selected female and male breeders in hapas (small enclosed semi rigid nets) (20 × 5 × 2 m). A total of 103 breeding hapas (2 × 1 × 1.5 m) were suspended in moderately saline water ponds (8–10 ppt) and in each hapa, one female was kept with one male. After seven days, fertilized eggs were collected from the mouth of the female and immediately transferred to hatching trays for artificial incubation. Each family was incubated in a separate hatching tray (40 × 20 × 10 cm) in low saline water (3–5 ppt) at 25–30 °C and pH 7.5–8.2. Once a full-sib family was successfully produced and a sufficiently large quantity of progeny (>600 fry per family) was attained, the male was then paired with a second female to produce paternal half-sib families. The mating pairs were mated again if they produced less 600 fry. A hierarchical nested mating design was applied, targeting one male mated to two females; not all the mate pairs were successful. As a result, there were 49 full-sib and 28 half-sib families. In the 2014 breeding season, a total of 77 families were produced from 63 male and 77 female brooders.

Fry rearing was conducted in nursery hapas (3 × 1.2 × 1.5 m with 2 mm mesh size) in a 1000 m^2^ low saline water (5–10 ppt) pond. The fry of each family were reared in three replicate hapas at an initial stocking density of 200 fry per hapa (or about 55 fry per square meter of surface water). For the first nursing period from hatching to 20 d, a commercial powder feed (45% protein AQUAXCEL®, Cargill Company) was provided four times daily with a feeding rate at 10% of the total body mass in hapas. The fry were transferred to a larger hapa (3 × 2 × 1.5 m) for further rearing from 21 d post hatching to tagging at 62 d post hatching. The feeding rate was reduced to 5% of total body weight and fry were fed twice a day (8.00 and 16.00) with a commercial pellet (35% protein). When the fingerlings reached 5–8 g, about 100 individuals were randomly sampled from each family (30–35 fingerlings per nursing hapa) and physically tagged with passive integrated transponder tags (PIT). The identification number, body weight (BW) and standard length (L) were recorded. The tagged fingerlings were then conditioned for 4–5 d in fibreglass tanks without feeding. During the conditioning period, fish that lost their tags or died were replaced by other individuals from the same family.

### Testing environments

After conditioning, the tagged fingerlings from 77 families were split into two groups (50 individuals per family in each group) and were randomly assigned to freshwater or moderate saline water (15–20 ppt) earthen ponds (1,500 m^2^ 30 × 50 × 1.5 m) located at Quy Kim Station in Hai Phong, 120 km from Hanoi. The salinity level in experimental ponds was controlled by adding freshwater (0 ppt) and sea water (30 ppt). The temperature and dissolved oxygen ranged from 17.3–34.5 °C and 3.5–6.4 mg/l in freshwater environment and from 17.8–34.7 °C and 3.7–6.5 mg/l in the moderately saline water environment. In both culture systems, the initial stocking density was 2.5 fish per square meter of surface water. During the grow-out period, the fish were fed a commercial dry pellet with 35% protein content twice a day (8.00 and 16.00) at the average feeding rate of 5% of the total body mass. The feeding rate was adjusted monthly according to the growth rate of the fish, the latter being determined from a random sample of 100 animals per environment. The same feeding, culturing and management practices were applied in both environments.

### Measurements

At the end of the grow-out period of approximately 120 d, all the experimental fish were harvested to measure body traits, sexual maturity, morphological deformity, gill condition and survival. Individual fish were weighted using a digital scale (nearest to 0.1 g). Standard length was also measured with a ruler (with precision of 1 mm). Body depth and width was measured at the highest vertical distance and thickness position, respectively, with a calliper (±0.1 mm). Survival was recorded as a binary trait and also computed from the difference between the number of fish at stocking and at harvest. Fish alive at harvest were coded as 1, otherwise as 0 for those that was dead (missing) at harvest. Maturity status of all individual fish was recorded as either: (i) immature and not ready to spawn with white/clear and flat genital papilla and normal abdomen (=0); and (ii) mature and ready to spawn with pink to red and protruding genital papilla, fully opened genital pore, and distended abdomen (=1). Morphological deformities commonly found in tilapia include: scale disorientation, gill, eyes, skeletal, soft tissue, and split fins deformity^18^. They were treated in the form of presence (abnormal) or absence (normal) and were coded as 0 = normal fish and 1 = morphological deformity (abnormal) fish (e.g. fin, body, operculum, eye and head deformities)^26^. Gill condition (red = 0; pale = 1 was an indicator of fish health related to parasite, bacteria or virus diseases) was recorded in all individual fish in both fresh and saline water. The total volume of feed which was used in each pond was recorded to calculate the FCR for individual fish with an assumption that all the fish in each environment had an equal chance to get the feed. FCR of individual fish in each environment was calculated:
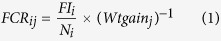
where ***FCR***_*ij*_ is feed conversion ratio of fish *j*^*th*^ in environment *i*^*th*^; ***FI***_*i*_ is total volume of feed in kilogram unit used in environment *i*^*th*^ (*i* = 1, 2); N_*i*_ is the average number of fish during the grow-out period in environment *i*^*th*^. ***Wtgain***_*j*_ is weight gain in kilogram unit of fish *j*^*th*^.

In addition, body condition or Fulton condition factor (**K**), which is the most common measurement to describe body shape of several fish species, was derived from measurements of body weight and standard length^4^. The K factor of each individual fish was calculated as described in the below equation by Casselman^27^:
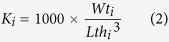
where ***K***_*i*_ is Fulton condition factor of fish *i*^*th*^; ***Wt***_*i*_ is the harvest weight in gram unit of fish *i*^*th*^; ***Lth***_*i*_ is the harvest length in centimetre unit of fish *i*^*th*^.

### Statistical analysis

Statistical analyses were performed on 26,295 data records collected between 2007 and 2014 in saline water together with 7,270 performance data in freshwater in 2014. The pedigree included six generations tracing back to the base population since 2007, comprising 36,145 individuals. The data was evaluated for normality before undertaking further analyses. Logarithmic, arcsine, square root or box-cox transformation was considered where appropriate. Exploratory analyses using a general linear model (GLM) were undertaken using PROC UNIVARIATE in SAS 9.3^28^.

After all significant fixed effects were identified (additional file 1); the final model fitted for all traits included the systematic effects of generation, testing environment, sex and their two-way interaction. Stocking weight and age from birth to harvest within sex and generation were fitted as linear covariates for body traits. The random effects are the additive genetics effects of individual fish and the maternal and common environment of dam. In matrix notation the mixed model was written as:
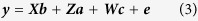
where **y** is the vector of observations for body traits, survival rate, maturity, FCR, deformity and gill condition, **b** is the vector of the fixed effects of generation (six generations and a base population pedigree), sex (female or male) and testing environments (saline- and fresh- water). Linear covariates of age from birth to harvest and stocking weight were fitted within sex and generation in the model. Vector **a** is the random animal additive genetic effects ~ (0, **A**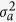) where **A** is the additive genetic (numerator) relationship matrix among the animals, **c** is the vector of dam effects (or maternal effects in addition to the additive genetics) ~ (0, **I**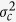) and ***e*** is the vector of residual effects ~ (0, **I**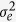). The dam component (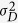) is most likely a combination of maternal and common environmental effects (thus, 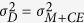, referred to as 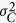) caused by the separate rearing of full-sib families until individuals reached a suitable size for physical tagging. **X**, **Z** and **W** are incidence matrices relating observations to fixed effects, additive genetic effect of the individual animal and common full-sib effect included in the model, respectively. Under the model [3], var(**a**) = **G** = **A**σ_a_^2^. The remaining effects are assumed to be distributed as var(**e**) = **R** = **I**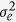, var(**c**) = **W** = **I**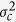, where **I** is an identity matrix. The expectations of all random effects are zero, cov (**a,e**) = 0 and cov (**a,c**) = 0 and thus var (**y**) = **ZGZ’**σ_a_^2^ + **WI**
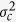
**W’ + R.**

The mixed model equation for the best linear unbiased estimator (BLUE) of estimable functions of **b** and the best linear unbiased prediction of **a** and **c** are:
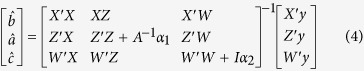
where α_1_ = 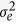/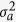 and α_2_ = 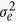/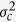.

*Heritabilities, phenotypic and genetic correlations*: For estimation of heritability and genetic correlations, the ASReml software version 3.0^29^ was used. In details: heritabilities were estimated from a single trait model. Phenotypic and genetic correlations were obtained from a series of bi- and tri-variate analyses, involving body weight (the selection criterion recorded on all animals) to avoid selection bias^30^.

First, measurements in both environments were treated as one trait, and the model included all the significant fixed and random effects as described in Equation 3. Second, trait measurements in fresh and saline water were treated independently in order to examine whether there are differences associated with the two culture environments. The model is as described above, also note that the fixed effect of environment was omitted from the statistical model. Genetic correlations between expressions of body traits or other characters studied in freshwater and moderate saline water ponds were estimated via numerator genetic relationships matrix **(A)**. Since the traits were measured on animals in different environments, there is no environmental covariance between them. The phenotypic correlations do not exist because any individual fish expressed the trait in only one environment.

Heritability for weight, body shape, FCR, survival and maturity were calculated as 
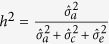
 and the maternal effect as 
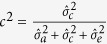
 where 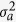 is the additive genetic variance, the maternal variance (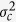) and the residual variance (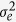). Genetic and phenotypic correlations among traits were calculated as the covariance divided by the product of the standard deviations of traits: 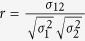 where 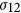 is the estimated additive genetic or phenotypic covariance between the two traits, and 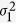
and 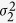
are the additive genetic or phenotypic variances of traits 1 and 2, respectively.

In addition to linear mixed model as described above (equation 3), survival, maturity and deformity were analysed by generalised linear mixed model (GLMM), assuming that the data followed a binomial distributions with a logit link function. Under the logit model, the link function (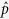 = e^x^/(1 + e^x^)) was used where *p* is the probability of fish survival (maturity or deformity) recorded at harvest. The fixed effects (*F*_*i*_) are the same as defined in Equation 3, except for the omission of sex when survival was analysed because this information was not known for dead or missing fish. The random effects are individual fish (*a*_*k*_) and dam (*d*_*l*_) for survival and maturity (Equation 6). Only the additive genetic effect of individual fish was fitted as a random term in the model for deformity. With this logit threshold model, heritability for survival and maturity was calculated using the variance of the logit link function, which implies a correction of the residual variance by factor π^2^/3.
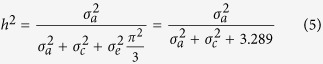
where 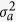 is the additive genetic variance, 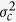 maternal and common environmental variance and 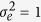

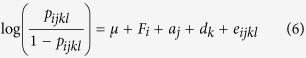


The maternal and common environmental variance 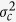 was not estimable for deformity, thus it was omitted from the above formula to calculate heritability for this traits. For binary observations (survival, maturity and deformity), estimates of heritability (*h*^*2*^) on the liability scales (logit) can be transformed to observed (0/1) scale using the formula of Robertson and Lerner^31^ as described by Thoa, *et al*.^25^. Due to only 0.016% of fish that had pale condition, heritability for this trait was not estimable with GLMM procedure as used for other binary traits. Linear sire model was employed to analyse this trait.

To test for significant differences in genetic parameter estimates between fresh and saline environments, we used the z-score as an approximate method for assessing whether heritability and correlation estimates were significantly different from each other or from zero, and whether genetic correlations between the two environments were significantly different from one^32^. The formula to calculate z-scores was as follows:
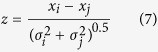
where *x*_*i*_ and *x*_*j*_ are the estimates of heritability, maternal effects, or genetic correlations for the two traits, and *σ*_*i*_and *σ*_*j*_ are their respective standard errors. Both *x*_*j*_ and *σ*_*j*_ were set to zero or one when we tested whether an estimate was significantly different from zero or one, respectively. Resulting z-scores were then tested against a large sample normal distribution.

### Ethics statement

All animal care and experimental procedures were performed in accordance with guidelines and regulations approved by the ethical committee of University of the Sunshine Coast, Queensland, Australia (ethical approval No. AN/S/13/29).

## Results

### Characteristics of testing environments and basic statistics for traits studied

The main difference between the two environments was the level of salinity (i.e. 0.74 ppt ± 0.07 in freshwater; 16.87 ppt ± 0.07 in saline water).The fish were reared at a starting weight of 4.5 g and a steady linear increase in growth over three sampling periods was recorded in both fresh and medium salinity environments (results not presented). At the final harvest (7270 fish measured at 196 d), the mean body weight of tilapia in moderate saline water was greater than those in freshwater (256.9 *vs.* 226.9 g, *P* < 0.05). Basic statistics for all traits are given in Table 1. Body traits generally had low standard deviations with one exception for body weight. Over six generations, the survival rate from stocking to harvest was 72.8%. The average maturity rate of this population was high (86.93%), whereas the proportion of fish having ‘pale’ gill condition was very low (0.016%). Deformity rate in both testing environments was only 0.148%. Feed conversion ratio, FCR (i.e. kg of food used to produce one kg of fish live weight) was 1.45 with high standard deviation (0.57).

### Trait differences between moderate saline- and fresh-water environments

Least squares means of traits studied (body weight, fitness, maturity, FCR and gill condition) obtained from a mixed model that included all possible systematic fixed effects (additional file 1) and random terms of individual fish and common full-sibs are shown in Table 2. The experimental fish reared in moderate saline water environment had significantly greater growth and lower FCR than those in freshwater whereas the opposite trend was found for body condition (Fulton condition or K factor) and survival rate. Maturation rate was 6% lower in moderately saline water than in freshwater (*P* < 0.001). The difference in deformity rate between the two testing environments was not significant (*P* > 0.05).

### Heritability and common environmental effects

Heritability (h^2^) and common environmental effects (c^2^) for traits studied (body weight, body condition, FCR, gill related disease, deformity, survival and sexual maturity) were estimated separately for each testing environment and across the two environments, using both animal and animal and dam models (Table 3). Across the environments, the estimates of heritability for harvest weight using the two models were moderate to high and significantly different from zero (*P* < 0.05). In general, heritability and common environmental effects for body traits was higher in saline water than in freshwater, but lower for body condition and survival rate. However, the differences in heritability for all traits studied between the two environments were not significant (*P* > 0.05). Moderate to high heritabilities for body condition and FCR were estimated by the animal model, whereas they were low and associated with high standard errors when the animal and dam model was used. Similar results were found for heritability of sexual maturity across the models. The heritability for survival was low to moderate (0.03–0.27) across the two environments. In addition, a very low heritability (0.01–0.07) was estimated for gill condition and deformity. For all traits except gill condition and deformity, the c^2^ effect ranged from 0.01 to 0.25 in both saline and fresh water. The large range of c^2^ indicates that by omission of this effect, the heritability for traits studied were overestimated.

### Genetic and phenotypic correlations among traits

Genetic and phenotypic correlations between traits studied were estimated separately in freshwater and moderate saline water (Table 4) and in the both environments (Table 5). Across environments, genetic and phenotypic correlations between body weight and FCR were very high and negative, whereas a positive and close to one genetic correlation was found between body weight and maturity. Genetic associations between body weight and fitness traits (survival and deformity) were not significant (Table 5). Similarly, there were low genetic correlations between body condition and the other traits studied. The genetic correlations of maturity rate with survival and deformity were moderate to high and negative, ranging from −0.60 ± 0.14 to −0.82 ± 0.06. On the other hand, survival was genetically correlated positively with sexual maturity (Table 5). Both genetic and phenotypic correlations among traits studied in saline water and freshwater environments (Table 4) had similar signs and magnitudes to those obtained across the two environment (Table 5).

### Genetic correlations between trait expressions in freshwater and moderate saline water

Table 6 shows genetic and common environmental correlations between the trait expressions in the two testing environments. The genetic correlations estimated for body weight, FCR, maturity and survival traits between freshwater and moderate saline water environments were very high and positive (*r*_*g*_ = 0.78 to 0.99, *P* < 0.05). The genetic correlation estimate for morphological deformity trait had a high standard error (0.99 ± 0.62). Similarly, common environmental correlations estimated for body weight, FCR and maturity were very high (0.87–0.99) (results not tabulated). The close to one genetic correlations for traits studied indicated that genotype by environment interaction was likely to be unimportant for many of these characteristics in the present population.

## Discussion

The principal aim of our study was to understand if the hypothesis proposed by Falconer^1^ is applicable to a tilapia population selected under medium salinity (i.e. less conducive for Nile tilapia), will produce genotypes which can perform well in both freshwater and saline water culture systems. We tested this by treating trait expressions in saline- and fresh-water as different characteristics and using multivariate analysis to estimate the genetic correlations for new traits, including sexual maturity, deformity and feed conversion ratio (FCR). The high and close to one estimates of genetic correlations obtained across trait expressions in saline- and fresh-water showed that the genotype by environment interaction (G × E) was not important for fitness (i.e. sexual maturity, deformity and survival) and growth related traits (i.e. feed conversion ratio and body weight) in the present population of Nile tilapia after six generations of selection. Our results indicate that the genetically improved line selected in saline water for high growth can be cultured successfully in freshwater systems. To the best of our knowledge, performance of the present salinity tolerance line has not been experimentally tested in prevailing environments that are conducive to tilapia culture, i.e. freshwater pond. Almost all studies in aquaculture species examined the G × E effect on body traits, with a few exceptions for fitness related traits, such as sexual maturity or deformity^22^,^23^. The genetic correlation for sexual maturity between freshwater and saline water environments was consistent with that in Rainbow trout (*Oncorhynchus mykiss*) shown by Kause, *et al*.^23^. In contrast, Wild, *et al*.^22^ reported a genotype by salt water interaction for early maturity in Atlantic salmon (*Salmo salar*). Across species, there are no published information on the G × E effect on feed conversion ratio (FCR) in aquaculture to compare with our current study.

In a comparison with the present study, in which the experimental fish were selected in a sub-optimal culture environment (i.e. moderate saline water of 15–20 ppt), other researchers used genetic lines selected under favourable environmental conditions and then conducted performance testing under culture systems that are generally close to the selection environment used in the nucleus. The literature^11^ suggest that the effect of genotype by environment interaction for production traits (body weight and survival) is unimportant when the selection and production environments are similar, such as freshwater ponds and cages^3^,^4^,^33^,^34^,^35^. These results are in good agreement with our high genetic correlations between the two environments for body weight (*r*_*g*_ = 0.92 ± 0.04) and for survival (*r*_*g*_ = 0.77 ± 0.03). By contrast, a significant G × E interaction occurred for harvest body weight when the selection environment in the nucleus differed greatly from production systems, such as freshwater *vs.* moderate saline water in Nile tilapia^5^, Arctic char (*Salvelinus alpinus*)^36^ and/or recirculating systems compared with pond in Sole (*Solea solea*)^37^. Similarly, Sylvén, *et al*.^38^ reported low genetic correlations in slaughter weight of Rainbow trout between fresh- and salt- (*r*_*g*_ = 0.72) and brackish water (*r*_*g*_ = 0.58). These results suggest that selection under favourable well-controlled environments in the nucleus not only result in re-ranking of breeding candidates but also a fraction of genetic gain achieved from the selection program can be captured in production systems. This contrasts with our findings showing that the present selected line under moderate saline water can capture about 77% and 92% genetic gain in survival and body weight when its offspring were grown under freshwater ponds. Our breeding program to improve growth performance under moderate saline water provides tilapia seed for both freshwater and saline water production systems. Conducting separate breeding programs may not be justified when the genetic correlation of the trait expressions between the two environments is greater than 0.7–0.8^39^.

In addition to the genetic correlations, we estimated heritability for a range of traits in saline- and fresh-water, separately. The non-significant difference in magnitude of heritability for these traits indicates that the G × E interaction was mainly due to scaling effect, i.e. there was no re-ranking of selected candidates between saline- and fresh-water. We also aimed to estimate heritability for new traits in tilapia, including deformity, sexual maturity, gill condition and FCR. Our first estimates of genetic parameters for fitness related traits show that there is an additive genetic component especially for sexual maturity (h^2^ = 0.32–0.4). This suggests that sexual maturity can respond to selection as reported by Wild, *et al*.^22^ in Atlantic salmon and Kause, *et al*.^23^ in Rainbow trout. Due to the very low proportion of animals that had deformity (0.15%) and ‘pale’ gill condition (0.016%) in this population, the heritability for these traits are not significantly different from zero, indicating that continuing collection of the data in future generations is needed in order to obtain reliable estimates for binary characteristics. Other studies show existence of useful additive genetic component for a range of deformity measures but the heritability for these traits was low to moderate and associated with high standard errors: 0.16–0.29 in European sea bass *Dicentrarchus labrax*^40^ and Yellowtail kingfish *Seriola lalandi*^41^.

Improving efficiency of food utilisation is crucial to reduce production costs of aquaculture enterprises. In aquatic species, it is costly and practically infeasible to record the feed intake of individual animal. Our calculation of feed conversion ratio (FCR) can be used as an approximate measure although it does not reflect a ‘real’ variation in feed efficiency among animals. However, this measure of FCR is practical under the conditions of commercial production. The present estimate of heritability indicates that FCR is heritable (h^2^ = 0.52–0.59 with animal model and 0.12–0.16 with animal and dam model). Using animal mixed model, Henryon, *et al*.^42^ studying rainbow trout reported a low additive genetic variation for feed conversion efficiency with a coefficient of additive genetic variation from 4.0–13.9%. Henryon, *et al*.^42^ reared each family separately in different tanks during the grow-out period whereas a communal rearing method was applied in our study. Similarly, low heritability for feed efficiency (0.03 ± 0.10–0.07 ± 0.11) was reported in European whitefish *Coregonus lavaretus*^43^ and Rainbow trout^44^.

After six generations of selection, our population continues to display large genetic variation for body weight in saline and freshwater environments with the estimate of heritability from 19 to 62%. The heritability obtained for body weight across the testing environments indicated that this population will continue responding to future selection. Heritability for body weight achieved in our study was higher than that reported previously in Nile tilapia^3^,^4^,^32^,^33^,^45^,^46^,^47^. In addition, estimates of heritability for survival (0.15 to 0.32) and body condition (0.06–0.25) in our study was in good agreement with those reported for survival^5^,^48^,^49^ and body shape of Nile tilapia^4^. It is important to note that, the above studies were conducted under freshwater condition with an exception in moderate saline water system by Luan, *et al*.^5^.

The third objective of our study was to test if the covariance between traits varies with culture environment. The difference in size and magnitude of genetic correlations are not significant between saline- and fresh-water environments. Furthermore, in tilapia, the genetic relationships among body traits, FCR, sexual maturity and deformity were not available. The negative genetic correlation between body weight and FCR indicates that fast growing animals selected for increased harvest body weight also converted feed more efficiently into growth. The close to one genetic correlation between weight and FCR is consistent with our assumption that animals in the same pond had equal access to feed and had a fixed amount of feed intake. Under a liberal feeding regime, body weight was moderately correlated genetically with FCR for Japanese flounder^50^, Rainbow trout^51^ and Coho salmon^52^.

Another important observation from our study is a high and positive genetic correlation (*r*_*g*_ = 0.84 ± 0.08) between body weight and sexual maturity. This suggests that selection for increased growth rate may lead to early maturation in Nile tilapia. Our results are consistent with those reported for other species, including Rainbow trout^23^ or Atlantic cod^53^. However, selection experiments for high production performance in aquatic animals generally showed both negative^54^,^55^ and positive^23^,^53^,^56^ effects on maturation. In a long term, a multi-trait selection approach using a desired gain (or restricted) index, as demonstrated in kingfish^41^, can improve traits showing antagonistic genetic correlations.

This is the first time, that genetic and phenotypic correlations between survival and deformity are reported in Nile tilapia. The high and negative genetic and phenotypic correlation (*r*_*g*_ = −0.82 ± 0.06) between survival and deformity is expected because deformed fish usually have lower survival abilities than healthy animal. Consistent with other studies^32^, we found that the body traits are controlled by the same set of genes as indicated by the close to one positive genetic correlations among weight, length, width and depth (results not shown). Interestingly, it is predicted that in the present population, selection for increased body weight can improve body condition (*r*_*g*_ between body weight and K factor = 0.19 ± 0.04). This is in contrast with the non-significant estimates between the two traits in other studies^4^.

The fish cultured in saline water showed better growth performance and efficiency of feed utilisation than those grown in freshwater (*P* < 0.001). The FCR estimate in our research (1.08–1.89) was within the range from 1.5 to 2.5 reported under practical commercial grow-out conditions in freshwater environments when using pelleted Nile tilapia feeds^57^. It is however not rigorous enough to compare the published information with our study because the experimental fish used in the literature are freshwater (e.g. GIFT strain) or high euryhaline capacity lines (e.g. Florida red tilapia), whereas our fish have undergone six generations of selection in a moderate saline water environment (15–20 ppt). The FCR is different among strains, life stages of growth development (larvae, fingerling and adult) and grow-out period/condition. Furthermore, the differences in type of feed and feeding approach could also lead to variation in FCR. In addition to the higher growth efficiency (high growth and low FCR) in the saline- than in fresh-water, the selected line had a delayed maturity in the former (saline water) relative to the latter (freshwater) environments that is favourable to commercial production. Early maturation has negative effects on growth and thus economic returns for fish producers, owing to early spawning and over density control in culture ponds. On the other hand, survival of the selected strain in this study was lower in moderate saline-than fresh-water environments.

In summary, we report for the first time the additive genetic variation and non-significant genotype by environment interaction effect for novel traits in Nile tilapia (sexual maturity, deformity, gill condition and feed conversion ratio) tested in both saline-and fresh-water systems. The moderate to high heritability for traits studied, especially body weight, indicates that our improved strain will continue responding to future selection. The close to one genetic correlations between trait expressions in the two testing environments indicate that our selected line under moderate salinity water can perform well in both saline-and fresh-water production environments, and that conducting two separate breeding programs for saline-and fresh- water is not justified for this population of Nile tilapia. Objective measurements of gonadal development of tilapia females and gill condition of live animals also merit further studies.

## Additional Information

**How to cite this article**: Thoa, N. P. *et al*. Does selection in a challenging environment produce Nile tilapia genotypes that can thrive in a range of production systems? *Sci. Rep.* **6**, 21486; doi: 10.1038/srep21486 (2016).

## Supplementary Material

Supplementary Information

## Figures and Tables

**Table 1:** Pedigree information and descriptive statistics for traits studied across the two testing environments.

Traits	Unit	Generation	N	Sire	Dam	Mean	SD	Min	Max
Weight	G	2007–2014	26,295	335	424	246.6	65.3	20.6	524
Length	cm	2007–2014	26,295	335	424	23.0	1.9	14.5	29.7
Width	cm	2014	6082	335	424	3.3	0.4	1.9	6.8
Depth	cm	2014	6082	335	424	7.4	0.8	4	10.3
Body condition	ratio	2007–2014	26,295	335	424	21.7	3.5	2.2	75.6
FCR	unit	2014	6082	77	77	1.45	0.57	0.48	5.31
Survival	%	2007–2014	36,145	335	424	72.8	44.5	0	1
Maturity	%	2014	7,270	77	77	87.5	33.71	0	1
Gill condition	%	2014	7,270	77	77	0.016	1.28	0	1
Deformity	%	2014	7,270	77	77	0.148	3.84	0	1

FCR = Food Conversion Ratio, Body condition or Fulton condition factor (K) = 1000 × [Weight(g)/Length^3^(cm)].

**Table 2:** Growth related traits (weight, length, width and depth), body condition, food conversion ratio, survival, sexual maturity and deformity of fish reared in saline water and freshwater environments in the latest generation in 2014 (Least squares means ± S.E).

Traits	Model	Saline water	Freshwater	Significant probability
Weight (g)	LMM	256.9 ± 1.09	226.9 ± 1.05	<0.001
Length (cm)	LMM	23.1 ± 0.03	22.5 ± 0.03	<0.001
Width (cm)	LMM	3.4 ± 0.01	3.2 ± 0.01	<0.001
Depth (cm)	LMM	7.5 ± 0.01	7.2 ± 0.01	<0.001
Body condition (ratio)	LMM	19.4 ± 0.04	20.4 ± 0.04	<0.001
Body condition (ratio)*	LMM	4.4 ± 0.009	4.7 ± 0.003	<0.001
FCR (unit)	LMM	1.08 ± 0.008	1.89 ± 0.008	<0.001
Survival (%)	GLMM	80.7 ± 0.66	86.6 ± 0.56	<0.001
Maturity (%)	GLMM	83.9 ± 0.69	89.5 ± 0.55	<0.001
Deformity (%)	GLMM	0.12 ± 0.06	0.17 ± 0.08	NS

NS = Non-significance, *Body condition also analysed on square root scale.

LMM = Linear Mixed Model and GLMM = Generalised Linear Mixed Model.

**Table 3:** Heritability (h^2^ ± S.E) and common environmental effects (c^2^ ± S.E) for traits studied in saline water (SW), fresh water (FW) and in both environments.

Traits	Environment	No. of records	Animal model	Animal + Dam model	
			h^2^	h^2^	c^2^
Weight	SW	23,109	0.57 ± 0.02	0.30 ± 0.02	0.22 ± 0.02
	FW	3,186	0.48 ± 0.07	0.19 ± 0.12	0.10 ± 0.05
	Both	26,295	0.62 ± 0.01	0.32 ± 0.04	0.25 ± 0.02
Body condition	SW	23,109	0.17 ± 0.02	0.08 ± 0.02	0.02 ± 0.004
	FW	3,186	0.28 ± 0.05	0.26 ± 0.11	0.01 ± 0.04
	Both	26,295	0.19 ± 0.02	0.09 ± 0.02	0.04 ± 0.005
FCR	SW	2,896	0.59 ± 0.08	0.12 ± 0.11	0.18 ± 0.06
	FW	3,186	0.52 ± 0.07	0.16 ± 0.13	0.13 ± 0.06
	Both	6,082	0.56 ± 0.07	0.12 ± 0.11	0.16 ± 0.05
Survival	SW	28,875	0.09 ± 0.01	0.15 ± 0.02	0.03 ± 0.01
	FW	7,270	0.01 ± 0.01	n.e.	n.e.
	Both	36,145	0.03 ± 0.006	0.17 ± 0.03	0.03 ± 0.01
	Both^*^	36,145	0.14 ± 0.01	0.04 ± 0.01	0.06 ± 0.01
Maturity	SW	2,896	0.40 ± 0.06	0.13 ± 0.09	0.09 ± 0.04
	FW	3,186	0.32 ± 0.06	0.06 ± 0.07	0.09 ± 0.04
	Both	6,082	0.36 ± 0.05	0.09 ± 0.07	0.08 ± 0.03
	Both^*^	6,082	0.25 ± 0.03	0.05 ± 0.06	0.18 ± 0.05
Deformity	SW	2,896	0.05 ± 0.03		
	FW	3,186	0.05 ± 0.03		
	Both	3,186	0.07 ± 0.02		
	Both**	6,082	0.53 ± 0.14	n.e.	n.e.
Gill condition	SW***	2,896	0.05 ± 0.02		
	FW***	3,186	0.01 ± 0.02		
	Both***	6,082	0.07 ± 0.02		

^*^Generalised threshold logistic animal and dam model, ** Generalised threshold logistic animal model, and ***Linear sire model, N.E. = non-estimable.

**Table 4:** Phenotypic (above the diagonal) and genetic (below the diagonal) correlations (SE in parenthesis) in saline- and fresh- water environments.

Traits	Saline water						Freshwater					
	Weight	BC	FCR	Survival	Maturity	Deformity	Weight	BC	FCR	Survival	Maturity	Deformity
Weight		0.17 (0.01)	−0.91 (.003)	0.05 (0.01)	0.44 (0.020)	−0.04 (0.02)		0.33 (0.03)	−0.92 (.004)	n.e.	0.42 (0.02)	−0.04 (0.02)
BC	0.22 (0.05)		−0.46 (0.03)	0.06 (0.01)	0.15 (0.03)	0.02 (0.02)	0.17 (0.14)		−0.38 (0.03)	−0.45 (0.070)	0.09 (0.03)	−0.03 (0.030)
FCR	−0.99 (0.01)	−0.46 (0.13)		−0.50 (0.02)	−0.47 (0.03)	n.e.	−0.93 (0.02)	−0.24 (0.13)		−0.66 (0.04)	−0.49 (0.02)	0.006 (0.02)
Survival	0.12 (0.11)	0.10 (0.06)	−0.76 (0.06)		−0.26 (0.03)	−0.32 (0.02)	n.e.	−0.10 (0.50)	−0.40 (0.26)		0.001 (0.03)	−0.33 (0.03)
Maturity	0.78 (0.12)	0.48 (0.14)	−0.77 (0.06)	0.38 (0.15)		−0.01 (0.02)	0.84 (0.05)	0.34 (0.13)	−0.85 (0.05)	0.10 (0.30)		−0.02 (0.02)
Deformity	−0.17 (0.28)	0.09 (0.34)	0.14 (0.28)	−0.82 (0.12)	−0.01 (0.29)		−0.23 (0.29)	−0.61 (0.15)	0.26 (0.29)	−0.97 (0.05)	0.05 (0.29)	

BC = Body condition and FCR = Food conversion ratio.

**Table 5:** Phenotypic (above the diagonal) and genetic (below the diagonal) correlations in both environments.

Traits	Weight	BC	FCR	Survival	Maturity	Deformity
Weight		0.21 ± 0.01	−0.90 ± 0.01	0.08 ± 0.01	0.50 ± 0.02	−0.04 ± 0.02
BC	0.19 ± 0.09		−0.50 ± 0.02	−0.43 ± 0.01	0.13 ± 0.03	−0.006 ± 0.02
FCR	−0.98 ± 0.04	−0.55 ± 0.10		−0.16 ± 0.03	−0.41 ± 0.01	0.05 ± 0.02
Survival	0.19 ± 0.04	−0.09 ± 0.11	−0.81 ± 0.05			0.05 ± 0.02
Maturity	0.84 ± 0.08	0.20 ± 0.15	−0.74 ± 0.14	0.58 ± 0.11		−0.34 ± 0.02
Deformity	−0.13 ± 0.18	0.28 ± 0.19	0.15 ± 0.17	−0.82 ± 0.06	−0.03 ± 0.17	

See Abbreviations in Table 4.

**Table 6:** Genetic (r_G_) correlations between the traits expressed in saline- (SW) and fresh-water (FW) environments.

Traits	Environment	Genetic correlation
Weight	Between SW and FW	0.92 ± 0.04
Body condition	Between SW and FW	0.97 ± 0.02
FCR	Between SW and FW	0.96 ± 0.08
Survival	Between SW and FW	0.77 ± 0.03
Maturity	Between SW and FW	0.96 ± 0.02
Deformity	Between SW and FW	0.99 ± 0.62^NS^

NS = Non-significance.

## References

[b1] Falconer D. Selection in different environments: effects on environmental sensitivity (reaction norm) and on mean performance. Genetical Research 56, 57–70 (1990).

[b2] Ninh N. H., Thoa N. P., Knibb W. & Nguyen N. H. Selection for enhanced growth performance of Nile tilapia (*Oreochromis niloticus*) in brackish water (15–20 ppt) in Vietnam. Aquaculture 428, 1–6 (2014).

[b3] Bentsen H. B. . Genetic improvement of farmed tilapias: Genetic parameters for body weight at harvest in Nile tilapia (*Oreochromis niloticus*) during five generations of testing in multiple environments. Aquaculture 338–341, 56–65 (2012).

[b4] Trọng T. Q., Mulder H. A., van Arendonk J. A. & Komen H. Heritability and genotype by environment interaction estimates for harvest weight, growth rate and shape of Nile tilapia (*Oreochromis niloticus*) grown in river cage and VAC in Vietnam. Aquaculture 384, 119–127 (2013).

[b5] Luan T. D., Olesen I., Ødegård J., Kolstad K. & Dan N. C. Genotype by environment interaction for harvest body weight and survival of nile tilapia (*Oreochromis niloticus*) in brackish and Fresh water ponds In the proceedings of the 8th International Symposium on Tilapia in Aquaculture 231–240 (Egypt, 2008).

[b6] Santos A. I. . Growth and survival rate of three genetic groups fed 28% and 34% protein diets. Aquaculture Research 45, 353–361 (2014).

[b7] Le Boucher R. . Genotype by diet interactions in European sea bass (*Dicentrarchus labrax* L.): Nutritional challenge with totally plant-based diets. Journal of animal science 91, 44–56 (2013). 23100583 10.2527/jas.2012-5311

[b8] Quinton C., Kause A., Ruohonen K. & Koskela J. Genetic relationships of body composition and feed utilization traits in European whitefish (*Coregonus lavaretus* L.) and implications for selective breeding in fishmeal-and soybean meal-based diet environments. Journal of animal science 85, 3198–3208 (2007). 17709787 10.2527/jas.2006-792

[b9] Ibarra, A. M. & Famula R. Genotype by environment interaction for adult body weights of shrimp *Penaeus vannamei* when grown at low and high densitie. Genet. Sel. Evol. 40, 541–551 (2008). 18694549 10.1186/1297-9686-40-5-541 PMC2674889

[b10] Fishback A. G., Danzmann R. G., Ferguson M. M. & Gibson J. P. Estimates of genetic parameters and genotype by environment interactions for growth traits of rainbow trout (*Oncorhynchus mykiss*) as inferred using molecular pedigrees. Aquaculture 206, 137–150 (2002).

[b11] Nguyen N. H. Genetic improvement for important farmed aquaculture species with a reference to carp, tilapia and prawns in Asia: achievements, lessons and challenges. Fish and Fisheries. 10.1111/faf.12122 (2015).

[b12] Kankainen M. . How to measure the economic impacts of changes in growth, feed efficiency and survival in aquaculture. Aquaculture Economics & Management 16, 341–364 (2012).

[b13] Verhaegen Y., Adriaens D., Wolf T. D., Dhert P. & Sorgeloos P. Deformities in larval gilthead sea bream (*Sparus aurata*): A qualitative and quantitative analysis using geometric morphometrics. Aquaculture 268, 156–168 (2007).

[b14] Castro J. . Heritability of skeleton abnormalities (lordosis, lack of operculum) in gilthead seabream (*Sparus aurata*) supported by microsatellite family data. Aquaculture 279, 18–22 (2008).

[b15] Tave D. Genetics for Fish Hatchery Managers . 2nd edn (Van Nostrand Reinhold, 1993).

[b16] Pasnik D., Evans J. & Klesius P. Development of skeletal deformities in a Streptococcus agalactiae-challenged male Nile tilapia (*Oreochromis niloticus*) broodfish and in its offspring. Bulletin-European Association of Fish Pathologists 27, 169 (2007).

[b17] Wargelius A., Fjelldal P. G. & Hansen T. Heat shock during early somatogenesis induces caudal vertebral column defects in Atlantic salmon (*Salmo salar*). *Development Genes* Evolution 215, 350–357 (2005). 10.1007/s00427-005-0482-0 15798920

[b18] Sun P. E., Hawkins W. E., Overstreet R. M. & Brown-Peterson N. J. Morphological deformities as biomarkers in fish from contaminated rivers in Taiwan. International journal of environmental research and public health 6, 2307–2331 (2009). 19742162 10.3390/ijerph6082307 PMC2738889

[b19] Leary R. F., Allendorf F. W. & Knudsen K. L. Effects of rearing density on meristics and developmental stability of rainbow trout. Copeia 1991, 44–49 (1991).

[b20] Krise W. F. & Smith R. A. Eye abnormalities of lake trout exposed to gas supersaturation. Prog Fish-Cult 55, 177–179 (1993).

[b21] Sawada Y. . Hypoxic conditions induce centrum defects in red sea bream Pagrus major (Temminck and Schlegel). Aquaculture Research 37, 805–812 (2006).

[b22] Wild V., Simianer H., Gjøen H.-M. & Gjerde B. Genetic parameters and genotype × environment interaction for early sexual maturity in Atlantic salmon (*Salmo salar*). Aquaculture 128, 51–65 (1994).

[b23] Kause A., Ritola O., Paananen T., Eskelinen U. & Mäntysaari E. Big and beautifu? Quantitative genetic parameters for appearance of large rainbow trout. Journal of Fish Biology 62, 610–622 (2003).

[b24] Meuwissen T. H. E. & Woolliams J. A. Effective sizes of livestock populations to prevent a decline in fitness. Theoret. Appl. Genetics 89, 1019–1026 (1994). 24178119 10.1007/BF00224533

[b25] Thoa N. P. . Genetic variation in survival of tilapia (*Oreochromis niloticus*, Linnaeus, 1758) fry during the early phase of rearing in brackish water environment (5–10 ppt). Aquaculture 442, 112–118 (2015).

[b26] Tave D. & Kim D. S. Review Article: Gross Abnormalities in Tilapia. Fisheries and Aquatic Sciences 14, 148–160 (2011).

[b27] Casselman J. M. Determination of age and growth In The biology of fish growth (eds Weatherley Alan H. & Gill H. S. ) Ch. 7, 209–242 (Academic Press, 1987).

[b28] SAS Institute. *SAS/STAT*^*®*^ *9.3. User’s Guide Release 9.3* . (SAS Institute Inc., NC, USA, 2011).

[b29] Gilmour A. R., Gogel B., Cullis B., Thompson R. & Butler D. ASReml user guide release 3.0. VSN International Ltd, Hemel Hempstead, UK (2009).

[b30] Kennedy B. W. Use of mixed model methodology in analysis of designed experiments in *Advances in Statistical Methods for Genetic Improvement of Livestock* (Springer-Verlag, Berlin, 77–97, 1990).

[b31] Robertson A. & Lerner I. M. The heritability of all-or-none traits; viability of poultry. Genetics 34, 395–411 (1949). 17247323 10.1093/genetics/34.4.395 PMC1209454

[b32] Nguyen N. H., Khaw H. L., Ponzoni R. W., Hamzah A. & Kamaruzzaman N. Can sexual dimorphism and body shape be altered in Nile tilapia (*Oreochromis niloticus*) by genetic means? Aquaculture 272, S38–S46 (2007).

[b33] Eknath A. E. . Genetic improvement of farmed tilapias: Composition and genetic parameters of a synthetic base population of *Oreochromis niloticus* for selective breeding. Aquaculture 273, 1–14 (2007).

[b34] Thodesen J. . Genetic improvement of tilapias in China: Genetic parameters and selection responses in growth of Nile tilapia (*Oreochromis niloticus*) after six generations of multi-trait selection for growth and fillet yield. Aquaculture 322–323, 51–64 (2011).

[b35] Khaw H. L., Ponzoni R. W., Hamzah A., Abu-Bakar K. R. & Bijma P. Genotype by production environment interaction in the GIFT strain of Nile tilapia (*Oreochromis niloticus*). Aquaculture 326–329, 53–60 (2012).

[b36] Chiasson M. A., Quinton C. D., Danzmann R. G. & Ferguson M. M. Comparative analysis of genetic parameters and quantitative trait loci for growth traits in Fraser strain Arctic charr (*Salvelinus alpinus*) reared in freshwater and brackish water environments. Journal of Animal Science 91, 2047–2056 (2013). 23478828 10.2527/jas.2012-5656

[b37] Mas-Muñoz J., Blonk R., Schrama J. W., van Arendonk J. & Komen H. Genotype by environment interaction for growth of sole (*Solea solea*) reared in an intensive aquaculture system and in a semi-natural environment. Aquaculture 410–411, 230–235 (2013).

[b38] Sylvén S., Rye M. & Simianer H. Interaction of genotype with production system for slaughter weight in rainbow trout (*Oncorhynchus mykiss*). Livestock Production Science 28, 253–263 (1991).

[b39] Mulder H., Veerkamp R., Ducro B., Van Arendonk J. & Bijma P. Optimization of dairy cattle breeding programs for different environments with genotype by environment interaction. Journal of dairy science 89, 1740–1752 (2006). 16606745 10.3168/jds.S0022-0302(06)72242-1

[b40] Dupont-Nivet M. . Heritabilities and GxE interactions for growth in the European sea bass (*Dicentrarchus labrax L.*) using a marker-based pedigree. Aquaculture 275, 81–87 (2008).

[b41] Nguyen N., Whatmore P., Miller A. & Knibb W. Quantitative genetic properties of four measures of deformity in yellowtail kingfish *Seriola lalandi* Valenciennes, 1833. Journal of fish diseases. 10.1111/jfd.12348 (2015). 25683477

[b42] Henryon M. . Genetic variation for growth rate, feed conversion efficiency and disease resistance exists within a farmed population of rainbow trout. Aquaculture 209, 59–76 (2002).

[b43] Cheryl D., Antti K. & Juha K. Breeding salmonids for feed efficiency in current fishmeal and future plant-based diet environments. Genet. Sel. Evol 39, 431–446 (2007). 17612482 10.1186/1297-9686-39-4-431 PMC2682821

[b44] Kinghorn B. Genetic variation in food conversion efficiency and growth in rainbow trout. Aquaculture 32, 141–155 (1983).

[b45] Nguyen N. H. . Correlated response in fillet weight and yield to selection for increased harvest weight in genetically improved farmed tilapia (GIFT strain), *Oreochromis niloticus*. *Aquaculture* 305, 1–5 (2010).

[b46] Ponzoni R. W. . Genetic improvement of Nile tilapia (*Oreochromis niloticus*) with special reference to the work conducted by the WorldFish Center with the GIFT strain. *Reviews in* Aquaculture 3, 27–41 (2011).

[b47] Hamzah A. . Genetic evaluation of the Genetically Improved Farmed Tilapia (GIFT) strain over ten generations of selection in Malaysia. Journal of Tropical Agricultural Science 37, 411–429 (2014).

[b48] Charo-Karisa H. . Heritability estimates and response to selection for growth of Nile tilapia (*Oreochromis niloticus*) in low-input earthen ponds. Aquaculture 261, 479–486 (2006).

[b49] Rezk M. A. . Selective breeding for increased body weight in a synthetic breed of Egyptian Nile tilapia, *Oreochromis niloticus*: Response to selection and genetic parameters. Aquaculture 293, 187–194 (2009).

[b50] Ogata H. Y., Oku H. & Murai T. Growth, feed efficiency and feed intake of offspring from selected and wild Japanese flounder (*Paralichthys olivaceus*). Aquaculture 211, 183–193 (2002).

[b51] Silverstein J. T., Hostuttler M. & Blemings K. P. Strain differences in feed efficiency measured as residual feed intake in individually reared rainbow trout, *Oncorhynchus mykiss* (Walbaum). Aquaculture research 36, 704–711 (2005).

[b52] Neely K. G., Myers J. M., Hard J. J. & Shearer K. D. Comparison of growth, feed intake and nutrient efficiency in a selected strain of coho salmon (*Oncorhynchus kisutch*) and its source stock. Aquaculture 283, 134–140 (2008).

[b53] Kristjánsson T. & Arnason T. Strong phenotypic and genetic correlation between size and first maturity in Atlantic cod *Gadus morhua* L. reared in commercial conditions. Aquaculture Research 46, 2185–2193 (2014).

[b54] Su G.-S., Liljedahl L.-E. & Gall G. A. E. Estimates of phenotypic and genetic parameters for within-season date and age at spawning of female rainbow trout. Aquaculture 171, 209–220 (1999).

[b55] Quinton C., Moghadasi S., McKay L. & McMillan I. Genetic parameters of body weight, female spawning date and age at sexual maturation in rainbow trout. Paper presented at *Seventh World Congress on Genetics Applied to Livestock Production: Session 06. Fish and shellfish breeding*, Montpellier, France: INRA. (2002, August 19-23).

[b56] Kolstad K., Thorland I., Refstie T. & Gjerde B. Genetic variation and genotype by location interaction in body weight, spinal deformity and sexual maturity in Atlantic cod (*Gadus morhua*) reared at different locations off Norway. Aquaculture 259, 66–73 (2006).

[b57] El-Sayed A.-F.M. On-farm feed management practices for Nile tilapia (*Oreochromis niloticus*) in Egypt. On-farm feeding and feed management in aquaculture (eds Hasan M.R. & New M.B. ). FAO Fisheries and Aquaculture Technical Paper No . 583, Rome, FAO 101–129 (2013).

