# Supplementary material for: Does selection in a challenging environment produce Nile tilapia genotypes that can thrive in a range of production systems?
Source: Sci Rep. 2016 Feb 19;6:21486. doi: 10.1038/srep21486 (PMC4759592; doi:10.1038/srep21486)
Supplement: Supplementary Information [file srep21486-s1.pdf]

Does selection in a challenging environment produce Nile tilapia genotypes that can thrive in a range of production systems?

Ngo Phu Thoa[a, b,*], Nguyen Huu Ninh[a], Wayne Knibb[b] and Nguyen Hong Nguyen[b,*]

[a]*Research Institute for Aquaculture No.1, Dinh Bang, Tu Son, Bac Ninh, Vietnam.*
[b]*Faculty of Science, Health, Education and Engineering, University of the Sunshine Coast, Maroochydore QLD 4558, Australia.*

*Corresponding author: Telephone +84 4 38273069; Fax +84 4 38273070;
Email address: npthoa@ria1.org and nnguyen@usc.edu.au

Additional file 1: Analysis of variance (F-statistic value and significant level)

Effect	DF	Weight	BC	FCR	Survival	Maturity	Gill	Deformity
Generation, G	5	123.0***	N.I	N.I.	273.4***	N.I	N.I	N.I
Environment	1	346.0***	689.5***	4778***	36.4***	33.3	0	0.48
Sex	1	1232.4***	3.2	1029***	N.I.	35.3***	0	0.00
Sex × Environment	6	259.3***	3.1	157.8***	N.I	36.3**	0	0.11
Age(Generation)	6	242.4***	37.2***	0.1	N.I	5.4*	0.11	0.44
Stock weight(G)	6	197.9***	2.4	82.9***	61.4***	118.0***	0.11	0.73

*P<0.05, **P<0.01 and ***P<0.001, N.I. = Not included in the model

BC = Body Condition, FCR = Food conversion ratio.

Generation was not included for body condition, maturity, gill and deformity because the data for these traits were collected from only one generation in 2014
